# Cognitive Reserve? Cognitive Capacity!

**DOI:** 10.3390/brainsci14121265

**Published:** 2024-12-17

**Authors:** Kenneth R. Paap

**Affiliations:** Department of Psychology, San Francisco State University, 1600 Holloway Avenue, San Francisco, CA 94132, USA; kenp@sfsu.edu

**Keywords:** cognitive reserve, cognitive capacity, processing resources

## Abstract

The concept of cognitive reserve (CR) has been a cornerstone in cognitive aging research, offering a framework to explain how life experiences like education, occupation, bilingualism, and physical exercise may buffer individuals from cognitive decline in the face of aging or neurological disease. However, this paper argues that the CR model, while influential, may have outlived its usefulness due to inherent limitations that constrain future research directions and unintentionally encourage “magical thinking”. Specifically, CR’s definition, which relies on cognitive performance being “better than expected” based on known measures of brain structure and function, makes the concept temporally bound to current scientific understanding, potentially stifling novel insights into cognition. In contrast, we propose a shift to a cognitive capacity (CC) framework, which views cognitive performance as being always determined by the brain’s structural and functional capacities, without needing to invoke expectations based on incomplete knowledge. The CC framework is broader, encompassing factors that either promote or demote cognitive performance by directly modifying brain structure and function. This reconceptualization opens avenues for investigating cognitive enhancement not only in the context of aging or disease but also in young, healthy individuals. By emphasizing causal pathways between brain changes and cognitive outcomes, this perspective provides a more flexible and testable approach to understanding the mechanisms behind cognitive performance and its modulation across the lifespan.

## 1. Cognitive Reserve? Cognitive Capacity!

Stern, Varangis, and Habeck [1] remind us in their opening sentence that “The concept of cognitive reserve proposes that specific life experiences result in more flexible or resilient cognitive processing allowing some people to cope better with age- or disease-related brain changes than others” p. 1. It is undoubtedly true that the banner of cognitive reserve (CR) has brought deserving attention to the potential benefits of cognitively stimulating activities in delaying or reducing the severity of cognitive decline. However, it may be the case that the concept of CR has outlived its usefulness because it is easily misunderstood and may constrain or bias the direction of future investigation. It (unintentionally) encourages magical thinking. Furthermore, as explained below, whether or not a factor is considered a CR factor depends on whether the compensatory changes in brain structure and function are known or unknown. 

My perspective will strike many readers as misguided given that the National Institute on Aging recently funded a Collaboratory on Research Definitions for Reserve and Resilience in Cognitive Aging and Dementia that invested substantial resources in formulating a framework for CR and resilience in aging. [2] Although the collaboratory intends and expects the common framework to facilitate progress in understanding the factors associated with successful aging, it also explicitly asserts that it has no intention to stifle creativity that strays from the framework. The NIA website states that “we want to encourage investigators who have different views or use a given concept differently to note how their definitions relate or differ with one of those described here”. In that spirit, consider the alternative framework offered later in this perspective.

### 1.1. The New Definition of Cognitive Reserve

The new NIA-sponsored framework for CR [3] is situated within a more general framework for resilience where resilience refers to the capacity of the brain to maintain cognition and function with aging and disease. Resilience is all about overcoming adverse circumstances.

The framework subcategorized resilience into three different mechanisms: CR, brain reserve, and brain maintenance. Brain reserve refers to a capacity for mitigating adversity simply because there is more or better brain structure and function to begin with. Individuals endowed with bigger brains can lose more gray-matter volume before a threshold for cognitive decline is reached. Brain maintenance refers to a capacity to slow the rate of brain degradation. CR includes an additional element as specified in this new definition:

“CR is a property of the brain that allows for cognitive performance that is better than expected given the degree of life-course related brain changes and brain injury or disease” [3]. 

The new element, highlighted with the underlining, is that an operational definition of CR will need to demonstrate that current cognitive performance is better than expected given the current measures of brain structure and function.

#### 1.1.1. Philosophy of Mind

If CR is a property of the brain (at molecular, cellular, or network levels) then the nature of CR depends on one’s philosophy of mind (i.e., one’s position on the relationship between the brain and cognition). Churchland’s [4] eliminative materialism assumes that the mind and behavior are determined by brain activity and, in fact, that the mind is completely explained by the brain’s neural networks and biochemical processes. Searle’s [5] biological naturalism also assumes that mental states are entirely caused by neurobiological processes, but allows that they are not necessarily reducible to those processes. For example, mental states may include higher-level (emergent) features. The key here is the common assumption that the only determinants of cognitive performance are underlying brain states. I assume that most of the scientific community hold views close to Churchland’s or Searle’s, that changes in cognitive ability are directly caused by changes in brain structure and function. This is also consistent with early discussions of CR. For example, in 2002 Stern observed that “From a strict point of view, the differences in cognitive processing envisioned by the cognitive reserve model must also have a physiologic basis, in that the brain must ultimately mediate all cognitive function” [6] (p. 451).

#### 1.1.2. A Conundrum

The definition of CR links cognitive performance to brain structure and function. However, there seems to be a disconnect in joining the components of this definition. If mental states are entirely caused by neurobiological processes, then how could cognitive performance be better than expected based on the state of these processes? The answer is that the definition ties CR to what is known, not to what is true. To oversimplify, consider this example. At a particular point in time the received view may be that cognitive performance is determined by total gray-matter volume. An insightful researcher believes that performance is also affected by resting-state functional connectivity (RSFC) between a set of key networks. Further assume that measures of RSFC account for variance in cognitive performance beyond that accounted for by gray-matter volume. By definition RSFC is a CR factor. Cognitive performance was better than expected based on gray-matter volume. But fame as a CR factor is fleeting because in any replication or extension RSFC is now expected (not unexpected as required by the definition) to affect cognitive performance.

#### 1.1.3. CR Proxies

Although CR is a property of the brain, the framework often focusses on proxies like education, occupational attainment, or bilingualism. In medical research a proxy measure is an indirect measure that is used to represent a variable of interest when direct measurement is not possible or practical. In contrast, in this context proxies are often the focus of interest because the researchers hope to accrue evidence that the proxy is an intervention that can promote brain structure and function. Replacing the previous assertation that CR is a property of the brain with a specific proxy, we might have: Bilingualism causes a change in brain structure or function that allows for cognitive performance that is better than expected given the degree of life-course-related brain changes and brain injury or disease. Thus, whether one focuses on a property of the brain or a proxy, one is confronted with a definition that places more emphasis on when something is known than what is true. Simply put, if one knows how bilingualism modifies the brain and how this modification enhances cognitive performance, then the measured performance is not better than expected. By definition, the proxy—bilingualism in this example—is no longer a CR factor. Despite what seems to me to be a straightforward logical implication of this new definition, it is notable that no one else has pointed out that when a neural account for the benefits bestowed by a CR factor (e.g., speaking two languages or playing improvisational jazz) becomes available, the operational definition requires that it no longer be considered a CR factor, even though we are now more confident than before that it contributes to resilience. 

## 2. A Cognitive Capacity Framework

**Figure 1:**
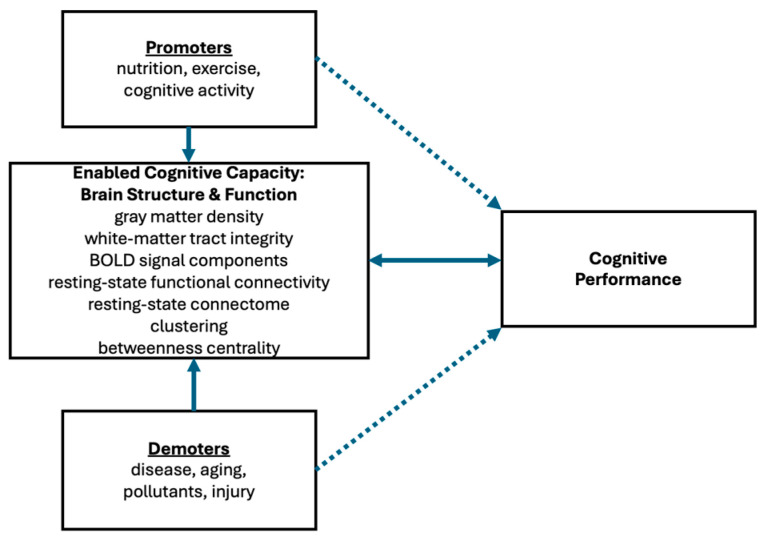
An alternative conceptual model that jettisons CR in favor of CC. Solid arrows represent causal pathways, while dashed arrows represent possible regression coefficients.

We have argued that it makes little sense to ask if cognitive performance is better than expected given the brain’s structure and functionality because it is the brain’s state, and nothing else, that determines the potential cognitive performance at any point in time. In contrast, it is important to know if a specified change in brain structure or function leads to an increase, decrease, or null effect on a targeted type of cognitive performance. Likewise, we would like to know how a myriad of factors indirectly effects cognitive performance by directly modifying brain structure and function. This framework is illustrated in Figure 1. The upper left box presents several examples of external factors that may promote better cognitive performance ranging from highly heritable head-starts (fluid intelligence) to physical health (nutrition, exercise), to cognitively stimulating activities such as education or thinking-occupations. On the flip side, there are factors that reduce cognitive capacity. The bottom left box presents external factors (e.g., disease, aging, or brain injury) that cause brain changes that demote cognitive performance. From a public health perspective there is high value in mapping the dose–response relationship of these factors and being able to predict the effects of the promoters and demoters (dashed-line arrows) on cognitive performance. Of course, critical opportunities for, say, pharmaceutical interventions will emerge from understanding the causal pathways from brain structure and function to cognitive performance. 

The CC framework assumes that cognitive performance is always determined by the cognitive capacity of the underlying brain structure and function and that our complementary task is to determine how it does so. Although we may not initially know how a new “promoter” is boosting cognitive performance there is no need to label it a CR factor. This is acknowledged in Stern’s (2002) original framework: “From a strict point of view, the differences in cognitive processing envisioned by the cognitive reserve model must also have a physiologic basis, in that the brain must ultimately mediate all cognitive function.” [6] p. 451.

The central box on the left of Figure 1 represents the structure and function of an individual’s brain. Its quality, in the aggregate and interactively, determines the maximum performance enabled by this brain. That is why the bold header reads Enabled Cognitive Capacity. If one thinks of each neuroscience measure as a parameter, then the current parameter values for any specific brain determine its processing capacity at this point in time. The current value of each parameter has been set by the accumulation of the promoters and demoters this individual has experienced. Cognitive neuroscientists can measure many of these facets of brain structure and function and several examples are listed. Studies can map the relationship between brain measures and cognitive performance, but this work is complicated by the distributed and interactive nature of the brain [7]. However, at a general level, it is reasonable to say that individuals differ in terms of the cognitive capacity currently enabled by their brain structure and function. That simple presumption has driven cognitive psychology since its inception. It is also reasonable to assume that individual capacities change over time as a result of exposure to or experiences with demoters and promoters. 

The new framework for CR and resilience presented in Stern et al. [3] requires a demoter (e.g., aging, disease). This deflects attention away from factors that more generally enhance brain structure and function, especially in young healthy brains. CR is characterized as a variable that influences the relationship between demoters and cognitive performance. The new CR framework acknowledges that this variable needs to modify brain structure and function such that it at least partially compensates for the demoter. This restricts the concept of cognitive reserve to factors that can counteract adversarial factors like aging and disease. Rather than characterizing CR as a variable that mitigates the effects of disease or aging, one can simply characterize CR factors as “promoters”, that is, as factors that enhance brain structure and function. This is a meaningful reconceptualization because it opens CR as a property of healthy brains as well as damaged brains. An individual who uses a brain network more efficiently or can switch to an alternate brain network has a cognitive advantage even if they have no brain pathology. One might replace cognitive reserve with a related concept called cognitive capacity (CC). This proposed amendment of the 2003 CR framework would not be a radical new idea. In his seminal paper, Stern [6] advocated that “…the concept of reserve should be extended to encompass variation in healthy individuals’ performance, particularly when they must perform at their maximum capacity”. 

### 2.1. What Is Cognitive Capacity as a Psychological Construct?

Following a long-standing tradition in cognitive psychology, one can think of brain structure and function as enabling cognitive capacity (CC) or, as it is also known, processing resources (George Miller [8], Daniel Kahneman [9], Don Norman [10], Mike Posner [11], Anne Treisman [12], Herb Simon [13], Chris Wickens, [14], Alan Baddeley [15]). 

#### 2.1.1. Kahneman’s Capacity Model

One idea, with a proud 50-year history, is that the efficiency of controlled processes depends on a limited supply of general processing capacity. For Kahneman [9], the terms “exert effort” and “invest capacity” are synonyms for “pay attention”. The specific capacity model Kahneman proposed has some interesting facets. One is that “The main assumption of the model is that the mobilization of effort in a task is controlled by the demands of the task, rather than by the performer’s intentions” p. 17. The corollary to this is that allocating additional mental effort (trying harder) has diminishing returns on performance. At this time there was also an interest in identifying distinctive physiological concomitants of effort, and Kahneman characterizes pupil size as providing a useful measure of the momentary exertion of effort. 

For present purposes, a limited capacity model of attention provides a reasonably straightforward mechanism for enhancing domain-general cognition—if all controlled processes compete for the same limited pool of processing capacity and if “exercise” increases the capacity of the reservoir, then far transfer from bilingualism to, say, nonverbal tasks should occur. To be careful, Kahneman did not speculate on the malleability of the total available capacity via an individual’s recent or cumulative use of effort. 

#### 2.1.2. Baumeister’s Strength Model

The Strength Model builds on the earlier processing-capacity models and especially focuses on the consequences of depleting resources: “self-regulation operates as if powered by a precious, limited resource akin to strength or energy” Thus, “After initial exertion of self-control or other top-down control, subsequent exertions in even seemingly unrelated contexts suffer”, Baumeister and Vohs [16], p. 113. This ego depletion effect has been found in many different contexts, but null results also frequently occur. Weak evidence for ego depletion undermines the Strength Model.

### 2.2. Behavioral Measures

One might note that there is a spirited debate regarding the existence of the depletion effect [17,18,19,20] that echoes the controversy surrounding the bilingual advantage in EF hypothesis [21].The first major meta-analysis of the depletion effect [22] included 198 comparisons and concluded that it was robust and medium in magnitude. However, no test for possible publication bias was reported until the individual-study data were shared with Carter and McCullough [17]. They reported substantial evidence for publication bias. When the bias was corrected using the PET method, the positive depletion effect was dramatically eliminated and numerically reversed. 

In an effort to adjudicate the existence of the depletion effect, Vohs et al. [23] conducted a preregistered multilaboratory project using what they dubbed a paradigmatic replication approach. Elements include recruiting domain experts to select and sanction the design and methods and recruiting statistical experts to develop and execute the analysis plan. The preregistered analysis found a nonsignificant result (d = 0.06) and a Bayesian analysis found that the data were four times more likely under the null than the alternative hypothesis. However, some non-preregistered tests were consistent with a small depletion effect, especially for participants who reported more fatigue. On the heels of the Vohs et al. multilab project, Dang et al. [24] published a multilab replication using the Stroop as a depleting task and an antisaccade as the outcome task. There was a small, but significant, ego depletion effect, d = 0.10 that increased to d = 0.16 after excluding participants performing at chance level on the outcome task. These results and all others led Baumeister and Tice [25] to conclude in their title that Ego Depletion is the Best Replicated Finding in All of Social Psychology. This conclusion is reasonable given the criteria set out, but it may reflect more the current status of social psychology than the robustness of the ego depletion effect. 

Baumeister and Vohs [16] propose that sustained effort enhances domain-general capacity. “One implication of the strength analogy is that it might be possible to increase self-regulatory capacity by exercise. Multiple studies have had people perform arbitrary exercise on self-control and then (after having practiced for a period of time) observed improvement on laboratory tests that involve behaviors quite different from the practiced ones.” p. 82. As long as it is assumed that all forms of top-down control (self-regulation, EF/cognitive control, decision making) draw capacity from the same limited pool, this framework predicts and accounts for experience-based improvements in domain-general cognitive ability. In contrast, Wickens [14] provides evidence for multiple pools. 

### 2.3. Capacity as Glucose

Baumeister and Vohs [16] speculate that the most likely physiological mechanism for capacity depletion and recovery is glucose dynamics. They review studies showing that consuming glucose benefits depleted people but not those in nondepleted conditions and that consuming equally tasty drinks without glucose provides no benefit. However, merely swishing the drinks around in the mouth for a few seconds also works, and the effect is immediate. Hagger and Chatzisaratis [26] cite evidence that brain regions associated with EF, such as the ACC, show increased activity in response to the glucose. This suggests that a mere taste of glucose can act as a physiological signal that more brain fuel is coming and that more resources can be allocated to the task at hand. 

Baumeister and Vohs [16] acknowledge that there is a question as to whether glucose itself enters the brain, but also observe that some neurotransmitters are made from it, thus rendering possible the view of glucose as “brain fuel”. However, Baumeister and Vohs are disinclined to treat glucose as a resource that is literally depleted during self-regulation. The brain does not run out of gas. Rather, ego-depletion effects may “…reflect the attempt to conserve what remains of the resource, rather than indicating a thorough going exhaustion of the supply” [16] p. 80. This resonates with the fact that although self-control usually leads to decrements due to ego depletion, people are able to overcome them if sufficiently motivated. Three ideas seem to fit together. First, that the self keeps track of how much capacity has been allocated (not how much is left); second, that the amount allocated might be signaled by the subjective experience of the mental effort expended; and third, that a fast pace of exertion usually leads to a reduction in the amount of capacity allocated to the current task. Thus, depletion effects are frequently observed, but not always. 

Baumeister and Vohs use an argument like this to keep the glucose hypothesis afloat while reducing expectations for a close relationship between glucose capacity and performance. “The central governor does not have the most important information, namely, how much glucose exists in storage around the body, so it uses various cues. Self-regulation does in fact depend on consuming a limited resource, and that people act as if their actions consume energy—even though their responses may be only weakly and indirectly linked to actual glucose consumption and reserves.” [16] p. 115. This framing of the role of glucose leads Baumeister and Vohs to suggest two possible mechanisms for experience to increase domain-general resources. The first is that one can learn that it is possible to continue allocating glucose for a longer period than previously believed. The second is that the physiological processes become more efficient with frequent use.

This revision of the strength model leads Vadillo et al. [27] to point out that the revised model is flexibly consistent with the results but is no longer grounded as a metabolic explanation. Furthermore, this group reported the results of a p-curve analysis [28] of 38 published tests of whether glucose becomes depleted as self-control depletes. A p-curve analysis speaks to the question of whether the statistically significant outcomes for a target research question are more likely to be false positives or true positives. Unlike a typical meta-analysis that includes all eligible effects, a p-curve analysis uses only those effects yielding *p* < 0.05. The number and magnitude of *p*-values greater than 0.05 are simply ignored. The logic is tied to the frequency distribution of the *p*-values of significant effects. If the true effect size is zero, then all *p*-values are equiprobable and the distribution of *p*-values in the analysis set should fall on a flat line. On the other hand, if there is a real effect, then the distribution will have a positive skew because large effects tested with powerful designs should yield very small *p*-values. For the set of studies analyzed by Vadillo et al., the results “follow a surprisingly flat distribution” and “suggest that the relationship between glucose levels and self-control behaviors might be unreliable”. With more agency, they conclude that “in light of these results, and pending further evidence, researchers and policymakers should refrain from drawing any conclusions about the role of glucose and self-control” [27] p. 1207.

### 2.4. EVC Model

Shenhav developed a model of attention allocation that assumes a limited information-processing capacity, but also provides a mechanism for how one decides to allocate that capacity [29,30]. Consider situations where an individual might decide to select one task from the available options. For any given option the specific task characteristics and the capacity demanded for peak performance will jointly determine what level of performance is attainable. The capacity actually allocated to the task determines the level of performance that will in fact be realized. Expected payoffs are weighed against the cost of exerting the associated levels of mental effort. For attractive options, the rewards will exceed the costs throughout a range of mental effort. In any case, the difference between rewards and costs is referred to as the expected value of control (EVC). The model enables the simulation of the process by which people consider the incentives and task demands associated with different options, and how much control they are willing to invest in each. 

The larger the potential benefit, the more willing a person is to perform a cognitively demanding task. A key to the question of enhancing self-control is the possibility that people learn from the outcomes of their efforts how much control to allocate in similar future situations. The degree to which one’s mental effort matters in determining performance is referred to as control efficacy. The degree to which performance matters in achieving long-term goals is referred to as performance efficacy. These constructs are potentially important, but it remains to be demonstrated whether any improvement through practice that enhances either control or performance efficacy are task-specific or domain-general. 

### 2.5. Individual Differences in Self-Control and Dopamine Synthesis Capacity

Cools’ [31] model of cognitive control emphasizes the need for a dynamic equilibrium between goal stabilization (viz., the ability to actively maintain and protect from distraction current goal representations) and destabilization (viz., the ability to allow new input to alter current goal representations). The mechanism is mediated by dopamine adjustments in either the prefrontal or striatal cortex. Cools cites evidence that dopamine potentiates the maintenance of neuronal firing in the prefrontal cortex that could, for example, stabilize goal representations in the working memory. Conversely, dopamine in the striatal cortex may potentiate the destabilization of goal representations needed for control flexibility. 

The work of Shenhav and Cools forms the basis for a recent study, [32], examining not only the relationship of the dopamine synthesis capacity to the working memory capacity (WMC), but also to trait impulsivity and spontaneous eye-blink rate (sEBR). The practical side of this work was to determine if, for example, a fast and inexpensive test of self-control ability (e.g., an impulsiveness scale) could work as an alternative to the expensive and invasive brain positron emission tomography (PET) scans needed to measure dopamine synthesis capacity. The theoretical interest to our present discussion is to explore the dopamine synthesis capacity as an individual difference that might account for enhancements in domain-general EF. Van den Bosch et al. [32] review tantalizing studies reporting associations between dopamine and WMC, trait impulsivity, and sEBR; but in their rigorous test with an unusually large sample (N = 94), none of these measures correlated significantly with the striatal dopamine synthesis capacity. This research group suggested that sample sizes of prior studies, with a median size of about 22, reported correlations that “might well have produced inflated and unstable estimates of the correlation coefficient” [32] p. 2. 

### 2.6. Increasing Bandwidth as Domain-General Enhancement

Perhaps more important to the question of enhancing domain-general EF is Shenhav’s proposal that capacity/effort does not have to be a form of “energy”. Alternatively, it can refer to a centralized mechanism that has a structural limit—a concept often used to describe the limits on the number of working memory representations that can be maintained and/or manipulated [33]. From this perspective, capacity is limited not for lack of energy, but rather to limit or prevent deleterious effects of cross talk in the processing system over which control presides. This alternative rejects mental effort as a limited resource, but not necessarily as a subjective experience. Mental effort may be aversive only to provide an index of the intrinsic and opportunity costs associated with an allocation policy. It enables the system to compute the EVC for each option and to make good choices. 

If the bottlenecks created by pathway overlap are so problematic, why not avert this problem by diminishing the shared use of representations: especially in the representational space provided by the human brain? Shenlav provides this interesting possibility. Shared representations support inference and generalization. Thus, the use of shared representations imposes a trade-off between its value for learning and abstraction on one hand and the constraints it imposes on the simultaneous execution of concurrent processing on the other. Decisions guided by the EVC can presumably find the sweet spot. 

### 2.7. Conditional Routing Model

Stocco and Prat have appealed to this increased bandwidth approach in their Conditional Routing Model for explaining bilingual advantages in domain-general EF. They hypothesized “that this enhanced capacity depends, at the neural level, on an increased capacity for controlling the transmission of signals to the prefrontal context through the basal ganglia” [34] p. 53. More specifically, they recorded neural activity, using fMRI, when bilinguals or monolinguals were engaged in rapid instructed task learning. The rules require keeping track of the operations that must be performed, in which order, and onto which variables. That is, on any given trial, three operations (e.g., Double, Half, Add) were presented, followed by a pair of target numbers (e.g., 2, 8) that needed to be mentally mapped and manipulated to yield a result (e.g., 4 + 4 = 8), and lastly compared to a probe (e.g., 3?). If the operations and target numbers change on every trial then, the variable bindings need constant updating. Bilinguals were faster than monolinguals when executing novel rules, and this improvement was associated with a greater modulation of activity in the basal ganglia. That is, only bilinguals showed different levels of activity in the basal ganglion in response to the degree of difficulty, indicating a greater capacity to recruit neural resources to cope with the increased difficulty. 

Stocco and Prat interpreted these results to mean that when facing increased demands for variable binding, monolinguals seem to employ a fairly constant pool of basal ganglia resources and consequently must take additional control steps (and time) to execute novel rules compared to practiced ones. In contrast, bilinguals seem to be able to recruit a larger set of basal ganglia, and thus, can manage a larger set of bindings in parallel, resulting in faster decision times. Stocco and Prat observe that “This interpretation may be viewed as differences in bandwidth, which determines the number of variables that can be routed during a single cognitive step in monolinguals and bilinguals” p. 59. 

### 2.8. Summary on Domain-General CC

The popularity of brain training suggests that many researchers resonate to the existence of a domain-general CC and the plausibility that it can be strengthened by the right type of practice (but see [35]). The cognitive and/or physiological mechanisms underlying such an increase in ability have not kept pace with our conceptual interests. The dominant approach treats attention as a limited resource that might expand with the right type of practice. This cognitive construct is sometimes embodied as an “energy” resource, such as glucose or dopamine. Associations between the estimated amount of metabolic capacity and individual differences in EF are not often measured, and when they are, the relationship is clearly not robust—in some cases it is completely absent. A second approach suggests that increases in volume and a greater capacity to modulate activity to adapt to task difficulty can “explain” domain-general increases in EF. 

### 2.9. Task-Specific Automaticity as CC

To this point, only domain-general mechanisms for CC have been considered. Domain-general CC can be applied to any task or situation, whereas domain-limited CC can be allocated only to a family of tasks. For example, a mathematician who can solve a problem using a different solution strategy that requires less WMC has additional capacity, but it is restricted to the domain of mathematics. The focus to this point has been on factors that can enhance domain-general CC. However, it is important to point out that the concept of CC can be expanded to include goal-directed behavior that relies heavily on automated processes. 

A core assumption in skill acquisition is that they are acquired in stages. Early in skill acquisition, our actions are often consciously guided, initiated intentionally, and demand mental effort. Later, one can act without deliberate intent, and with far less effort—because one becomes more reliant on specialized routines that are unique to the task, and directly link internal and external states to actions. This coincides with Posner’s [36] distinction between automatic and controlled processing, whereby the former is unconscious, triggered without intention, and requires little or no processing capacity. It also describes the transition from controlled to automatic processing, as articulated by Chein and Schneider [37]. The simple point here is that all cognitive skills (e.g., typing, driving, playing the violin, or air traffic control) become more automatic with practice. Thus, repeated practice or training on any task requiring specialized cognitive skill can be considered a promoter for that cognitive ability, and the underlying changes to brain structure and function are likely to involve representational plasticity in the regions and networks required by the task. 

As described above, there is a reciprocity between resource-demanding cognitive control and resource-light automatic control because the growth of skill automaticity reduces reliance on executive functions, working memory, and attentional resources, which are more susceptible to aging. Automatic skills tend to be more resilient to aging than those relying on the control mechanisms located in the prefrontal cortex [38,39].

To summarize, the CC framework depicted in Figure 1 covers the quality of goal-directed behavior generated from either controlled or automatic processing. The former is more important for the community of researchers studying aging because executive functioning is required in many facets of everyday life, and they are far more vulnerable to the adverse effects of aging. 

### 2.10. Echoes of Doubt in the Cognitive Reserve Canyon

The action editor brought to my attention several recent publications that raise similar issues about the logic and utility of the traditional CR framework. A perspective article published by Kremen and associates [40] at UCSD echoes several sympathetic points. Perhaps most importantly, they endorse constructs very similar to those I introduced in Figure 1. What I refer to as enabled cognitive capacity (CC), Kremen et al. refers to as total neural resources: “... the sum total of a dynamic process that involves the effects of genes, accumulated experiences throughout life, and adaptability…”. Both frameworks emphasize that current brain structure and function determine peak cognitive performance (CP) at any point in time. Both approaches assume that a CC embodied in brain structure and function determines CP, but a fissure emerges when Kremen et al. refer to CP as cognitive reserve and CC as brain reserve. They point out that the dictionary definition of “reserve” fits the general concept of a supply of general-purpose resources. However, in general, it also carries a connotation of an amount reserved for a special purpose, and in the context of cognitive aging, it is loaded with this baggage. Although they clearly assert that their use of cognitive reserve does not imply “better than expected” performance, this admonition is likely to be ignored or quickly forgotten. In summary, the Kremen et al. framework relies on an easily confused distinction between cognitive reserve and cognitive resilience that can be avoided by replacing CR with just-plain CC. The Kremen et al. article raises many interesting issues. For example, their discussion of reverse causation and its potential role in the research on cognitive aging is excellent. However, they do not discuss the potential of extending the CC construct to healthy brains or discuss the potential mechanisms by which the brain may adapt to have an increased cognitive capacity. 

In their critique of CR, Nilsson and Lövdén [41] tip their hand in their title: “naming is not explaining”. They argue that the current framework for CR does not allow for a testable theory because CR cannot be operationally defined when it is conceptualized as the residual variance in cognitive performance not explained by the available measure of brain structure and function (see their Figure 3c,d). They infer that an individual has CR because they also adopt the assumption that cognition and action are completely explained by brain activity. Thus, if the current set of measures of brain structure and function accounts for only part of the individual’s cognitive performance, the remainder can be labeled CR, but “naming is not explaining”. Nilsson and Lövdén go on to cogently observe that, “The lack of an operational definition is unsatisfactory because it means, paradoxically, that the measure of the cognitive reserve concept does not depend on the construct validity of the concept itself, but on the validity of the operational definition of brain integrity” p. 5. To reiterate my view in this context, why retain the concept of CR as a brain state that is “better than expected”? As illustrated in Figure 1, we want to continue to evolve more and better measures of brain integrity (its structure and function) that determines the embodied CC underlying cognitive performance and concomitantly adds to the list of external factors that either promote or demote CC. We also need to come to a better understanding of the mechanisms that lead to increases or decreases in CC. The concept of CR, as inferred from better-than-expected CP, may be worse than a red herring, as it creates the logical conundrum described in the introduction and, as Nilsson and Lövdén point out, leave us with a concept that cannot be operationally defined. 

This perspective started with a discussion on the recent definition for CR funded and endorsed by the NIA: a property of the brain that allows for cognitive performance that is better than expected given the degree of life-course related brain change and brain injury or disease. I argued, as illustrated in Figure 1, for a more complete and straightforward framework for studying the relationship between the brain and cognition, which is that the CC (enabled by the structure and function of the brain) determines CP, and we need to validate the set of external factors that promote or demote CP through changes to CC. Ironically, much of the cognitive aging community has adopted this strategy in practice despite holding on to the term “cognitive reserve”. This will be demonstrated with two quite recent examples: a meta-analysis by Opdebeeck et al. [42] and a systematic review by Panico et al. [43]. 

The Opdebeeck et al. meta-analyses assesses “the associations between the proxy measure of cognitive reserve (CR) and cognition…. As CR cannot be directly measured, it is commonly indexed by those experiences and activities thought to increase it” p. 1. The proxies included educational level, occupational status, and engagement in cognitively stimulating activities. The cognitive domains included memory, EF, visuospatial ability, and language. The effect size of interest was the mean r between a proxy measure of CR and a measure of CP. For example, the mean r for the 57 studies reporting the association between educational level and EF performance was r = 0.29. This result, which could be restated as a correlation between a potential promoter (educational level) and EF, was r = 0.29. Most of the 135 studies in the complete meta-analyses did not measure any aspect of brain structure or function. Note that these studies have nothing to do with assessing the degree to which individuals are performing better-than-expected, given their brain integrity. Calling the measure of educational level “CR” is misleading. Asking whether higher educational levels are associated with greater CC as indexed by a measure of cognitive performance is not. 

The recent systematic review of “the relationship between cognitive reserve and cognition in healthy adults” by Panico et al. (2023) was based on 13 published studies that met their criteria. Only one was included in the Opdebeeck et al. meta-analysis. However, both studies examine the relationship between “CR and general cognitive functioning”. Once again, CR is measured as the degree to which a participant has experienced a CR proxy, such as years of education. The key result is the relationship between the CR proxy and a measure of CP. As such, the observations made about the Opdebeeck et al. meta-analysis carry over to this systematic review. The predictor (e.g., years of education, IQ score, lifestyle questionnaire) assesses the degree to which the proxy predicts some type of cognitive performance but has nothing to do with whether it would be better-than-expected given the participant’s brain integrity. It may be worthwhile to repeat my warning that retaining the term CR is misleading and unnecessary, as the goal of these studies is to determine if some activity or experience has a positive effect on CP. If it does, and if one can confidently rule out confounds and faulty reverse inferences, then confidence is gained that this factor is a promoter of CC. Finally, one might note that the correlations in this systematic review are somewhat weaker and inconsistent than in the preceding meta-analysis. 

Pappalettera et al. [44] offer the newest critique of the conceptual and operational definitions of CR. For them, CR is determined by the capacity and efficiency of the brain. Capacity refers to the ability of the brain to activate neural networks to a progressively higher degree as task difficulty increases. This subtly differs from the enabled CC in the framework illustrated in Figure 1 because CC includes all aspects of brain structure and function that contribute to cognitive performance. In contrast, Pappalettera et al. separated efficiency from capacity, whereby efficiency refers to performing a cognitive task to criterion with less activation. As discussed earlier, my CC framework considers multiple mechanisms for enhancing CC in the broad sense, and this includes enhancing the pool of processing resources (like Pappalettera’s capacity) and also expanding bandwidth (like their efficiency). They emphasize the distinction between brain structure and function and define CR as an individual’s ability to maintain or regain cognitive function despite brain aging, damage, or disease. But if that ability is determined by the underlying brain structure and function, it is not clear what role CR plays beyond the constructs already represented in Figure 1. 

## 3. Conclusions

The concept of CR has contributed to an enhanced understanding of how life experiences can delay or mitigate the impacts of age-related cognitive decline. However, our evolving understanding of cognition fosters a shift from the traditional CR framework to one that emphasizes CC—a more general and flexible construct. CR’s reliance on the notion of “better than expected” cognitive performance, based on the current understanding of brain structure and function, inadvertently limits its utility. The alternative framework of CC offers a more direct and measurable approach, focusing on understanding how various external factors—both promoters and demoters—affect brain structure and function, which in turn determines cognitive performance.

By acknowledging the full range of factors that enhance or degrade cognitive function across the lifespan, and focusing on understanding the causal mechanisms by which these factors affect brain structure and function, the CC framework moves beyond the limitations of CR. It also opens the door to studying how the brain can maximize performance across a wide array of contexts, including in healthy individuals. The transition from a CR-based approach to a CC-based model allows for the inclusion of both brain structure and function as determinants of cognitive potential, without restricting this understanding to only adverse contexts such as aging and disease.

By focusing on CC rather than CR, researchers can explore more deeply the structural and functional mechanisms that underlie cognitive performance, leading to more effective interventions and strategies for cognitive enhancement in both healthy and impaired populations. This broader framework may ultimately provide a clearer path for future research, expanding our understanding of cognitive performance and its modulation across the lifespan.
